# Relationships Between D-Dimer Levels and Stroke Risk as Well as Adverse Clinical Outcomes After Acute Ischemic Stroke or Transient Ischemic Attack: A Systematic Review and Meta-Analysis

**DOI:** 10.3389/fneur.2021.670730

**Published:** 2021-06-07

**Authors:** Bing Yuan, Tong Yang, Tao Yan, Wenke Cheng, Xiancong Bu

**Affiliations:** ^1^Department of Neurology, Zaozhuang Municipal Hospital, Zaozhuang, China; ^2^Department of Hyperbaric Oxygen, Zaozhuang Municipal Hospital, Zaozhuang, China; ^3^Department of Emergency, Taierzhuang District People's Hospital, Zaozhuang, China; ^4^Department of Cardiology, Heart Center Leipzig at University Leipzig, Leipzig, Germany

**Keywords:** D-dimer, stroke, mortality, poor functional outcomes, meta-analysis

## Abstract

**Objective:** Abnormal elevation of D-dimer levels is an important indicator of disseminated intravascular clotting. Therefore, we hypothesized that high D-dimer levels were associated with the risk of stroke and adverse clinical outcomes of patients with acute ischemic stroke (AIS) or transient ischemic attack (TIA).

**Methods:** The present meta-analysis aimed to systematically analyze the associations between D-dimer and the risk of stroke as well as the clinical outcomes of patients with post-stroke or TIA. Meanwhile, dose–response analyses were conducted when there were sufficient data available. Three electronic databases including Pubmed, the Embase database, and the Cochrane Library were searched by two investigators independently. All the pooled results were expressed as risk ratios (RRs).

**Results:** Finally, 22 prospective cohort studies were included into this meta-analysis. The results suggested that high D-dimer levels were associated with increased risks of total stroke (RR 1.4, 95%CI 1.20–1.63), hemorrhagic stroke (RR 1.25, 95%CI 0.69–2.25), and ischemic Stroke (RR 1.55, 95%CI 1.22–1.98), and the dose-dependent relationship was not found upon dose–response analyses. Besides, the high D-dimer levels on admission were correlated with increased risks of all-cause mortality [RR 1.77, 95% confidence interval (CI) 1.26–2.49], 5-day recurrence (RR 2.28, 95%CI 1.32–3.95), and poor functional outcomes (RR 2.01, 95%CI 1.71–2.36) in patients with AIS or TIA.

**Conclusions:** On the whole, high D-dimer levels may be associated with the risks of total stroke and ischemic stroke, but not with hemorrhagic stroke. However, dose–response analyses do not reveal distinct evidence for a dose-dependent association of D-dimer levels with the risk of stroke. Besides, high D-dimer levels on admission may predict adverse clinical outcomes, including all-cause mortality, 5-day recurrence, and 90-day poor functional outcomes, of patients with AIS or TIA. More studies are warranted to quantify the effect of D-dimer levels on the risk of stroke or TIA, so as to verify and substantiate this conclusion in the future.

## Introduction

Cerebrovascular disease is the second cause of death and disability worldwide. According to statistics, ischemic stroke (IS) and transient ischemic attack (TIA) account for 70% of cerebrovascular diseases in China (1). Currently, acute ischemic stroke (AIS) is mainly diagnosed based on medical records, neurological examination, and brain imaging (CT or MRI) (2). In patients with AIS, a small infarct at the early stage may only result in a mild headache and numbness in the extremities. However, these minor symptoms are often ignored by the patient, leading to a further increase in infarct size. With the increase in the infarct size, the patient may experience slurred speech, restricted limb movement, or even blurred consciousness. For patients with early acute cerebral infarction, they may only present with a mild headache and numbness in the limbs; however, with the further expansion of the infarct area, the neurological symptoms of patients may worsen sharply, resulting in impaired consciousness or even sudden death. Therefore, in such critical situations, past medical history cannot be accurately obtained for most patients, which has posed a great challenge for the early disease diagnosis, and even experienced neurologists have difficulties in estimating the condition and predicting patient outcomes (3).

D-dimer is considered as a marker of intravascular protein degradation, which is ascribed to the action of coagulase (factor IIa), issue XIIIa, and fibrinolysin (4, 5). In clinical practice, abnormal elevation of D-dimer levels is an important indicator of disseminated intravascular clotting, and the low plasma D-dimer levels can effectively rule out some critical thromboembolic events, like deep venous thrombosis (DVT) and pulmonary embolism (PE) (6). Although D-dimer levels have been clearly shown to be associated with long-term mortality in the general population (7), only a few studies have assessed the correlations between D-dimer levels and the risk of stroke (8, 9) or the adverse clinical outcomes in post-stroke patients (10, 11).

Therefore, we hypothesized that high D-dimer levels were associated with the risk of stroke and the adverse clinical outcomes of patients with post-stroke or TIA and that differences in D-dimer level might be statistically significant between the stroke subtypes. To this end, based on existing studies, we performed a meta-analysis to systematically analyze the associations between D-dimer and the risk of stroke and the clinical outcomes of patients with post-stroke or TIA, and dose-response analyses were conducted if there were sufficient data available.

## Methods

The protocol and report of this meta-analysis were conducted based on the meta-analysis of observational studies from epidemiological guidelines (12). Only data from published studies were extracted in this meta-analysis, so ethical approval was not required.

### Literature Retrieval and Study Selection

Two investigators (Yuan and Yang) independently searched three electronic databases including Pubmed, the Embase database, and the Cochrane Library from inception to December 15th, 2020, so as to identify the eligible studies published in the English language. Besides, the reference lists in relevant studies were also searched manually. To ensure a broad search, three sets of medical subject headings (Mesh) were adopted, namely, “D-dimer,” “Stroke,” and “Transient Ischemic Attack.” The Boolean operator “OR” was employed for “Stroke” and “Transient Ischemic Attack,” “AND” was used for “D-dimer” and “Stroke/Transient Ischemic Attack,” and “OR” was used in each group. In addition, the potentially available meta-analyses and systematic reviews were also comprehensively reviewed. The detailed search strategy is presented in Appendix 1.

The study inclusion criteria were as follows: (1). the study population was the general population aged >18 years with new onset of stroke or TIA; (2). the exposures of interests were the D-dimer levels; (3). the study endpoints were the risk of stroke or TIA, mortality, recurrence, and poor functional outcomes; (4). the study design was limited to prospective studies (prospective cohort studies or randomized controlled trials RCTs); and (5). studies with available maximum adjusted odds ratios (ORs), relative risk (RRs), hazard ratios (HRs), and corresponding 95% confidence intervals (CIs). Meanwhile, the study exclusion criteria were shown below: (1). the study population suffered from specific diseases such as cancer and cardiovascular disease, or the non-stroke patients; (2). the exposures of interests were not the D-dimer levels; (3). the study outcomes did not report the risk of stroke or TIA, mortality, recurrence, or poor functional outcomes; (4). the study design was not prospective studies; (5). the maximum adjusted ORs, RRs, HRs, and corresponding 95% CIs were not reported or obtained; and (6). reviews, case reports and letters were excluded.

### Data Extraction and Quality Assessment

Two investigators independently searched relevant articles by their titles and abstracts. Data including first author, publication year, country, sample size, numbers of female/male cases, mean age, time of recruitment, follow-up time, D-dimer level, type of stroke, stroke ascertainment, and endpoints were extracted using a uniform data list. Any difference between the two investigators was resolved by consultation or the opinion of a third investigator. In addition, the Newcastle–Ottawa Scale (NOS) was adopted to assess the quality of observational studies, with a total score of nine stars (13). Studies with a NOS score ≥ 6 stars were considered as high-quality studies, while those with a NOS score <6 stars as low-quality studies.

### Statistical Analysis

Due to the differences in D-dimer levels reported between studies, we converted D-dimer levels to ng/ml. The adverse clinical outcomes analyzed included the risk of mortality, recurrence, and poor functional outcomes [defined as the modified Rankin Scale (mRS) ≥3].

In this study, the primary endpoint was qualitative analysis on the relationships between D-dimer levels and the risk of stroke and adverse clinical outcomes after acute stroke or TIA (including the risk of mortality, recurrence, and poor functional outcomes). To be specific, the impacts of different D-dimer levels on the risk of stroke and adverse clinical outcomes after acute stroke were systemically analyzed by comparing the high level to normal and low levels. Generally speaking, HRs were roughly equal to RRs (14). In addition, to use more available data, when the incidence rates of study outcomes in the overall population and subgroup populations were <10% or unavailable, ORs were approximated to be equal to RRs (15). Otherwise, ORs were converted into RRs according to the formula RR = OR/[(1-P_0_) + (P_0_ × OR)], where P_0_ indicates the incidence rate of the outcome in the unexposed group (16). Meanwhile, the corresponding 95%CIs were converted by the following formula, SElog (RR) = SElog (OR) × log (RR)/log (OR) (17). All the pooled results were expressed as RRs. Furthermore, we also applied the *I*^2^ statistic in evaluating the possible heterogeneities among studies, with *I*^2^-values of 25, 50, and 75% indicating low, moderate, and high heterogeneities, respectively. Besides, if the pooled results included a total number of over 10 studies, subgroup and meta-regression analyses were conducted to further explore the potential sources of heterogeneity between studies. Moreover, sensitivity analysis was also performed by eliminating one study each time to examine the effect of one study on the pooled results. To more conservatively estimate the pooled RRs, we adopted the random-effect model, since it explained well the heterogeneity between studies. Besides, Begger's-tests were performed to assess the publication bias (18).

The secondary endpoint of this study was the quantitative analysis on the relationships of higher D-dimer levels with the risks of stroke and adverse clinical outcomes. In parallel, the dose–response relationship was assessed. To this end, dose–response analyses were conducted based on the theory proposed by Xu and Doi (19). Specifically, in this “one-stage” framework approach, each of the included studies was considered as a cluster across the whole population, which required that the study should include at least two categories. Moreover, a method was applied in the restricted cubic splines to fit the potential non-linear trends at three nodes, and the non-linear *p*-values were calculated by testing the second spline coefficient to zero. Typically, a non-linear model was applied upon *p* ≤ 0.05; otherwise, a linear model was adopted. Further, the average of the upper and lower bounds was taken as the midpoint for each D-dimer category, and then the respective RRs were assigned to each midpoint. Meanwhile, in the case of open-study interval, the amplitude was assumed to be identical to that of the adjacent category (20). All statistical analyses were completed using the Stata 12.0 software.

## Results

**Figure 1:**
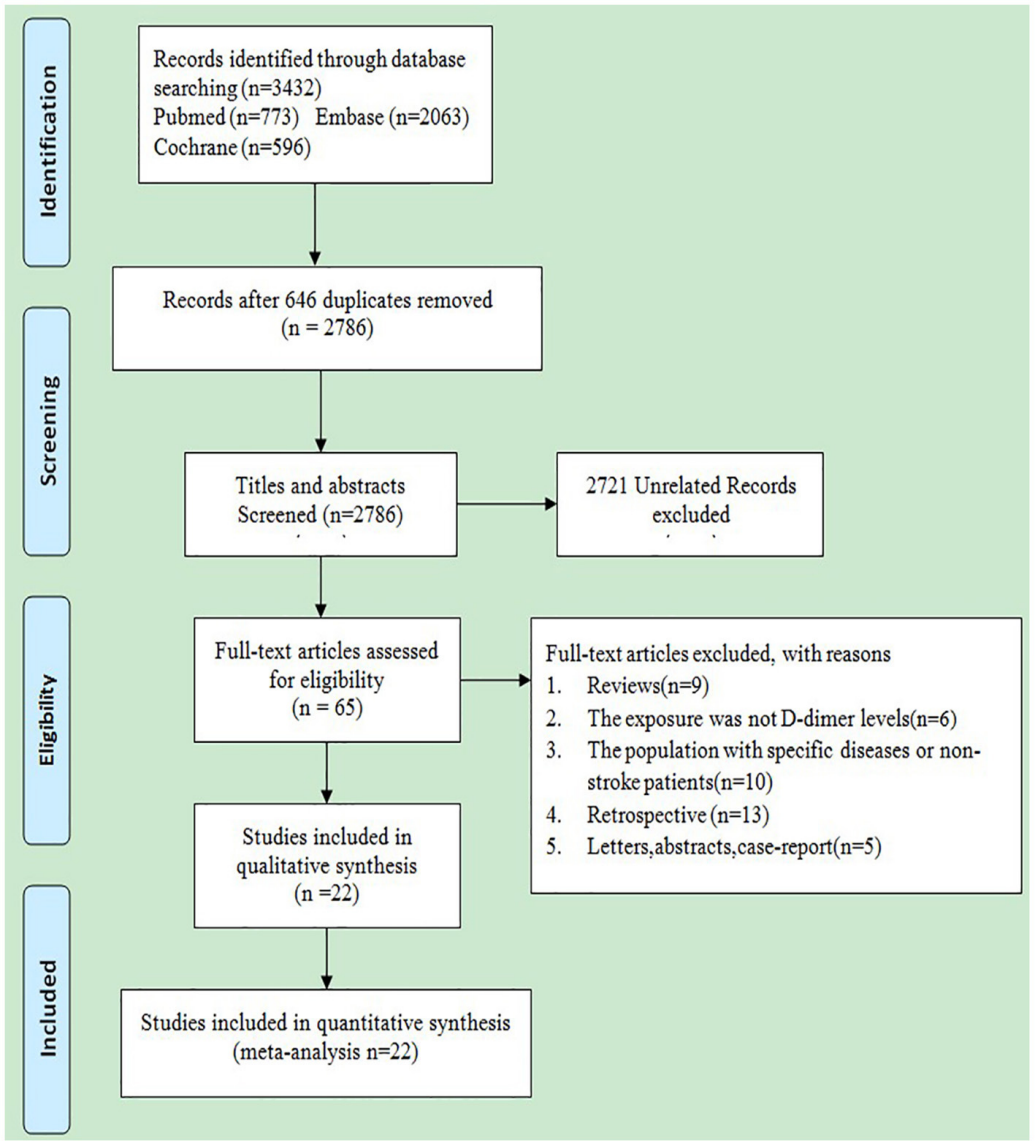
Flowchart of search results.

Altogether, 3,432 studies were identified from the three electronic databases (PubMed, Embase database, and Cochrane library), as shown in Figure 1. No additional study was identified by manual search. Among these 3,432 studies, 2,786 were retained after removing 646 duplicates; then, 2,721 irrelevant studies were further eliminated by screening titles and abstracts. Thereafter, the full texts of the remaining 65 studies were carefully read, among which 42 were excluded as a result of (1) reviews (*n* = 9); (2) the exposure being not D-dimer levels (*n* = 6); (3) the population suffering from specific diseases or the non-stroke patients (*n* = 10); (4) retrospective studies (*n* = 12); and (5) letters, abstracts, or case reports (*n* = 5).

Finally, 22 prospective cohort studies (3, 8, 9, 21–39) were included into this meta-analysis. Zakai et al. reported the correlations between D-dimer levels and the risks of stroke and hemorrhagic stroke (HS); therefore, it was analyzed as two studies separately (26, 27). The detailed baseline characteristics of the enrolled studies are presented in Table 1. The average age of the population in most of the included studies ranged from 60 to 75 years, and D-dimer collection was completed within 24 h in all patients with AIS or TIA. Among the 22 included studies, nine involving 27,044 participants mentioned the associations between D-dimer levels and the risk of stroke, and the study population was only the stroke patients, whereas 13 studies involving 14,435 participants reported the associations between D-dimer levels and the risk of adverse clinical outcomes in patients with AIS or TIA. To be specific, two out of these 13 studies involving 10,614 patients (*n* = 2 AIS) reported the all-cause mortality; two enrolling 426 patients (*n* = 2 AIS) mentioned the 30-day mortality; three recruiting 12,206 patients (*n* = 2 AIS, *n* = 1 AS or TIA) reported the 90-day mortality; two involving 203 patients (*n* = 2 AIS) mentioned the 5-day recurrence on diffusion-weighted imaging (DWI); and eight involving 13.710 patients (*n* = 5 AIS, *n* = 3 AIS or TIA) reported the 90-day poor functional outcomes. As shown in Supplementary Table 1, all the enrolled studies had a quality score above six stars and were considered as high-quality studies.

**Table 1:** The detailed characters of the included 22 prospective studies.

**References**	**Country**	**Sample size**	**Female/male**	**Mean age**	**Time of recruitment**	**Follow-up**	**D-Dimer range**	**D-Dimer assay**	**Type of stroke**	**Stroke status/ascertainment**	**Endpoints**
**Before stroke**											
Raffield et al. (21)	USA	4,163	2,585/1,578	55 y	2000–2004	4 y	0.01–11.98 ug/ml	ITA	Total stroke	Medical records	Total stroke, all-cause mortality
Smith et al. (22)	UK	2,208	0/2,182	56.9 y	1984–1988	Median 13 y	<9 ng/ml; 9–17 ng/ml; >17 ng/ml	ELISA	IS	ICD	IS
Tzoulaki et al. (23)	UK	1,592	783/809	64.9 y	NR	Mean 17 y	68–146 ng/ml	ELISA	Total stroke	Medical records	Total stroke
Carcaillon et al. (24)	France	1,254	759/495	74 y	1999–2001	4 y	NR	ELISA	IS, HS	CT or MRI	IS, HS
Wannamethee et al. (25)	UK	3,358	0/3,358	68.7 y	1978–1980	Mean 9 y	<56.8 ng/ml; 56.8–97.4 ng/ml; >97.5 ng/ml	ELISA	Total stroke	CT or MRI	Total stroke
Folsom et al. (9)	USA	11,415	6,632/4,783	59.8 y	1992–1995	Median 18 y	≤0.14 ug/ml; 0.15–0.22 ug/ml; 0.23–0.32 ug/ml; 0.33–0.49 ug/ml; >0.49 ug/ml	ITA	Total stroke, IS, HS	ICD-9	Stroke; IS; HS
Di Castelnuovo et al. (8)	Italy	822	550/282	49.4 y	1993–1998	35–71 y	<100 ng/ml; 100–127 ng/ml; 127.1–166 ng/ml; >166 ng/ml	ITA	Total stroke, IS, HS	ICD-10	Total stroke; IS; HS
Zakai et al. (26)	USA	1,180	664/516	65.4	2003–2007	Median 5.8 y	0–0.22 ug/ml; 0.23–0.32 ug/ml; 0.33–0.51 ug/ml; 0.52–0.89 ug/ml; ≥0.9 ug/ml	ITA	Total stroke	Medical records	Total stroke; Per-SD
Zakai et al. (27)	USA	1,052	530/522	65.1	2003–2007	Median 5.8 y	0.08–0.29 ug/ml; 0.29–0.62 ug/ml; 0.62–2.64 ug/ml	ITA	HS	Medical records	HS
**After stroke**											
Squizzato et al. (28)	Italy	96	54/42	74.9 y	1998.1–1999.12	61.5 months	≤5 ug/ml; 0.51–1.5 ug/ml; >1.5 ug/ml	ITA	AIS or TIA	Post-stroke/CT	All-cause mortality
Uestuendag et al. (3)	Turkey	91	49/42	64.5 y	2007.2–2007.12	10 months	≤5 ug/ml; 0.51–1.5 ug/ml; >1.51 ug/ml	ITA	AIS	Post-stroke/CT or MRI	30 d-mortality
Abdel Ghani et al. (29)	Egypt	50	17/33	60.6 y	NR	5 days	NR	ITA	AIS	Post-stroke/CT or MRI	5 d-DWI recurrence
Park et al. (30)	South Korea	175	80/95	66 y	2009.1–2010.6	90 days	355–1629 ng/ml	ELISA	AIS	Post-stroke/CT or MRI	90 d-poor functional outcomes
Sato et al. (31)	Japan	130	44/86	66–81 y	2011.10–2017.2	90 days	600–2,800 ug/l	NR	AIS or TIA	Post-stroke/CT or MRI	90 d-poor functional outcomes
Liu et al. (32)	China	1,468	508/960	64.26 y	2016.4–2019.12	1 y	≤0.5 mg/l; >0.5 mg/l	ELISA	AIS	Post-stroke/CT or MRI	90 d-poor functional outcomes; 90 d-mortality
Hou et al. (33)	China	10,518	3,283/7,235	62.3 y	2015.8–2018.3	1 y	<0.6 ug/ml; 6–1 ug/ml; 1.1–2.0 ug/ml; >2.0 ug/ml	ITA	AIS or TIA	Post-stroke/CT or MRI	90 d-poor functional outcomes; 90 d-mortality; all-cause mortaltiy
Shibazaki et al. (34)	Japan	335	125/210	72.3 y	2006.3–2008.4	30 days	0–9.5 ug/ml	NR	AIS	Post-stroke/CT or MRI	30 d-mortality
Yang et al. (35)	China	220	93/127	Median 68y	2011.2–2012.12	90 days	0.35–4.62 mg/l	ITA	AIS	Post-stroke/MRI	90 d-poor functional outcomes; 90 d-mortality
Kang et al. (36)	Korea	153	54/99	64.6 y	2004.12–2006.3	5 days	220–1,190 ng/ml	ELISA	AIS	Post-stroke/MRI	5 d-DWI recurrence
Yao et al. (37)	China	877	287/600	Median 64 y	2017.1–2018.8	90 days	≤0.24 mg/l; 0.25–0.56 mg/l; 0.57–1.78 mg/l; >1.78 mg/l	ITA	AIS	Post-stroke/CT or MRI	90 d-poor functional outcomes
Whiteley et al. (38)	UK	268	156/112	74.4 y	2007.3–2009.2	90 days	109–440 ng/ml	ELISA	AIS	Post-stroke/CT or MRI	90 d-poor functional outcomes
Sienkiewicz-Jarosz et al. (39)	Poland	54	27/27	73.3 y	NR	90 days	NR	ITA	AIS	Post-stroke/CT or MRI	90 d-poor functional outcomes

*AIS, acute ischemic stroke; TIA, transient ischemic attack; CT, computed tomographic; ITA, immunoturbidimetric assay; MRI, magnetic resonance imaging; ELISA, enzyme-linked immunosorbent assay Long-term Mortality, mortality over 3 months; 30d-mortaliy, mortality at 30 day; 30d-Poor Functional Outcomes, poor functional outcome at 30 days; 90d-Poor Functional Outcomes, poor functional outcome at 90 days; 5d-DWI recurrence, recurrence on 5-day diffusion-weighted; HS, haemorrhagic stroke; IS, ischemic stroke; ICD-9, International Classification of Diseases, Ninth Revision; ICD-10, ICD-9, International Classification of Diseases, tenth Revision; Per-SD, Per standard deviation Increment*.

## Meta-Analysis

### Pre-Stroke

#### Total Stroke

**Figure 2:**
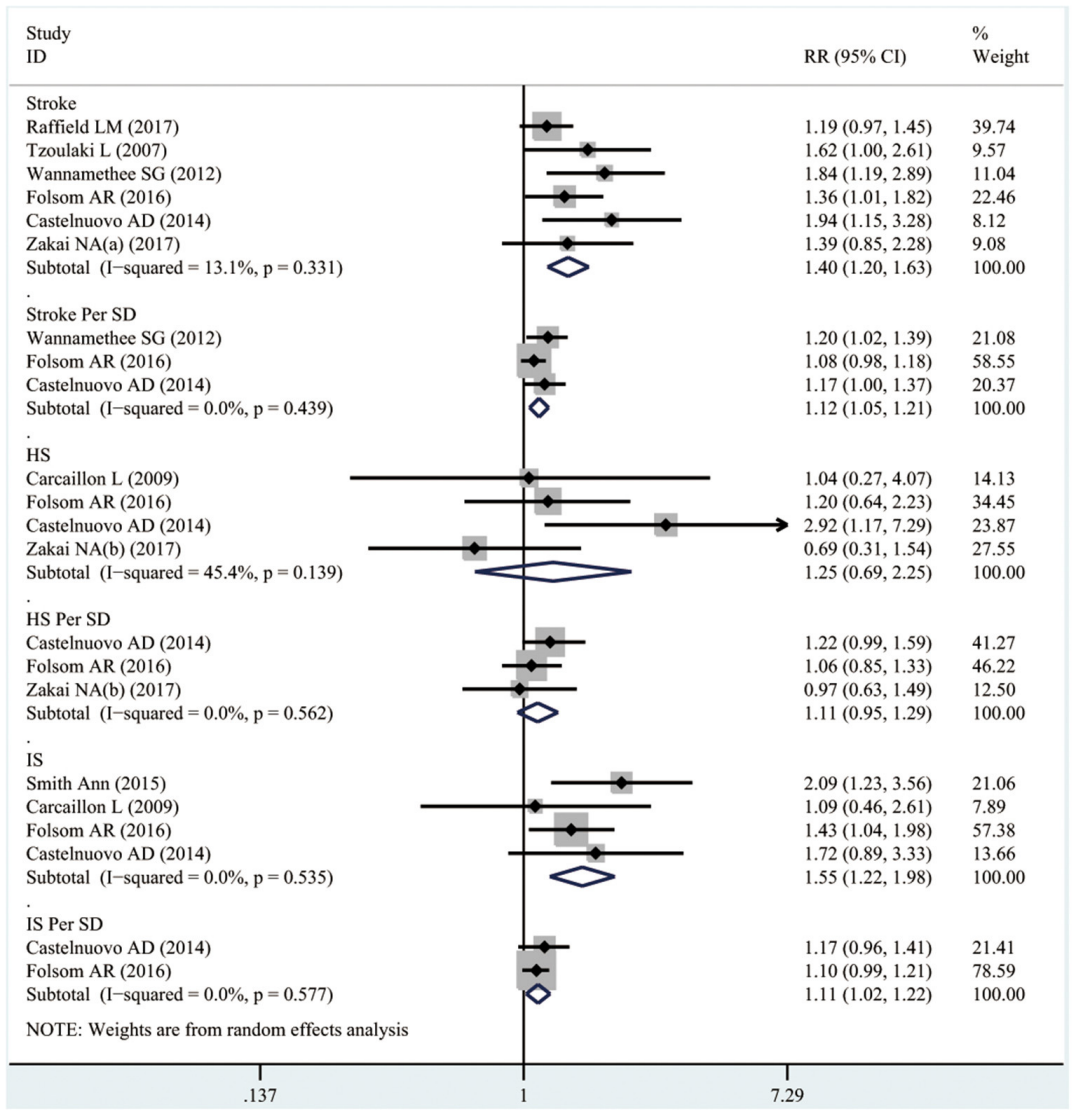
The RRs of high D-dimer levels for the risk of total stroke, IS, and HS.

As shown in Figure 2, six studies involving 22,590 patients mentioned the associations between high D-dimer levels and the risk of stroke. As a result, high D-dimer levels significantly increased the risk of total stroke by 40% (RR 1.4, 95%CI 1.20–1.63; *I*^2^ 13.1%). Similarly, the risk of total stroke increased by 12% with the increase in per standard deviation (per-SD) increment of D-dimer level (RR 1.12, 95%CI 1.05–1.21; *I*^2^ 0%).

**Figure 3:**
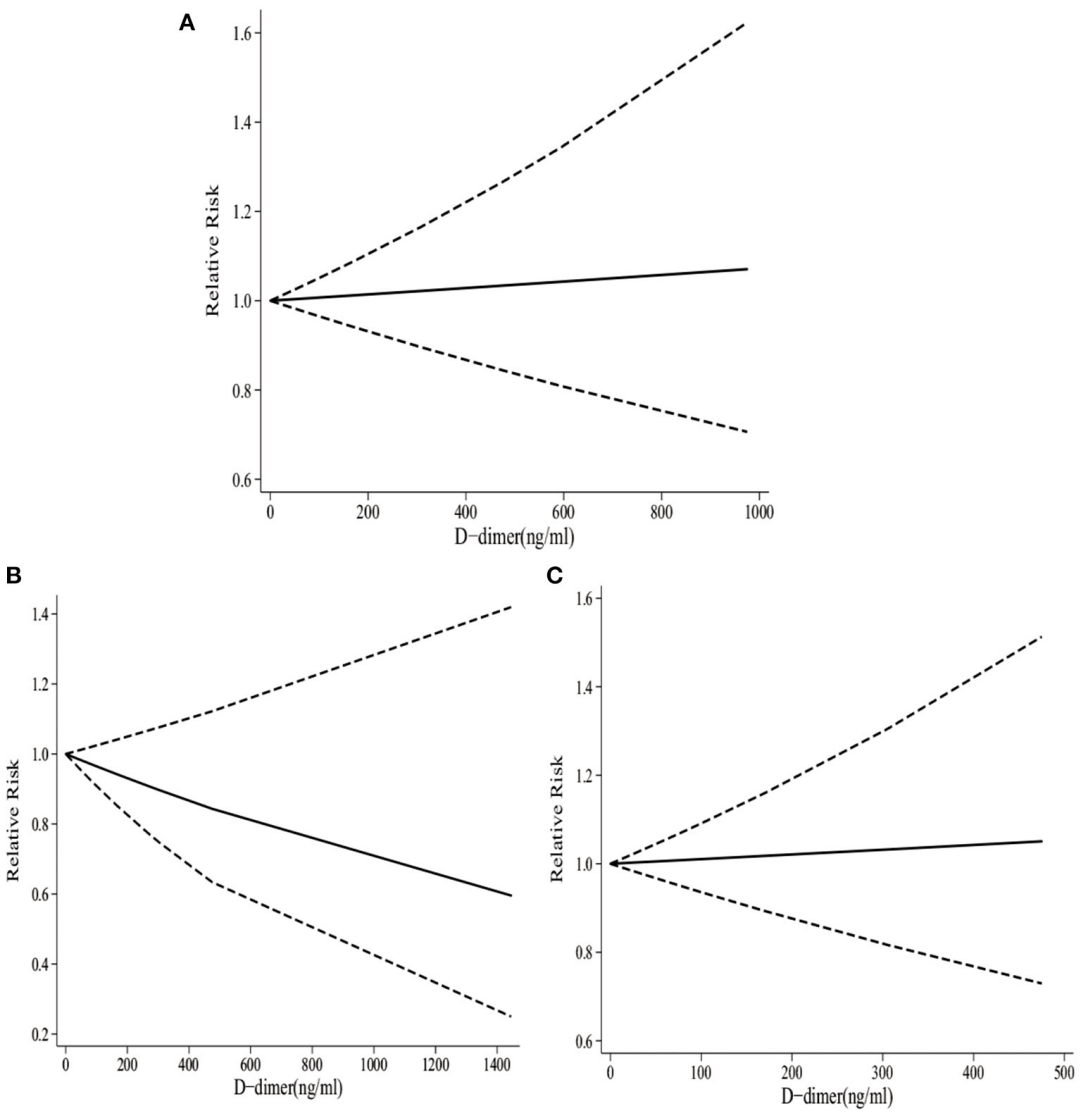
The dose–response of D-dimer levels and total stroke, IS, and HS. **(A)** The dose-response of D-dimer levels and total stroke. **(B)** The dose-response of D-dimer levels and IS. **(C)** The dose-response of D-dimer levels and HS.

It was illustrated from Figure 3A that there was a linear relationship between D-dimer levels and the risk of stroke (*p*_*non*−*linearity*_ = 0.51). Specifically, dose–response analyses on four studies indicated that D-dimer levels showed a weak positive correlation with the risk of stroke. When the D-dimer level increased by 50 ng/ml, the risk of stroke increased by 0.3 % accordingly (RR 1.003, 95% CI 0.969–1.039).

#### Hemorrhagic Stroke

As presented in Figure 2, four studies involving 14,543 participants reported the associations between high D-dimer levels and the risk of HS. There was no obvious evidence supporting that the high D-dimer levels were associated with the increased risk of HS (RR 1.25, 95%CI 0.69–2.25; *I*^2^ 45.4%). Similarly, the risk of HS did not increase with the per standard deviation (per-SD) increment in D-dimer level (RR 1.11, 95%CI 0.95–1.29; *I*^2^ 0%).

According to dose–response analyses on three studies shown in Figure 3B, there was a linear relationship between D-dimer and the risk of HS (*p*_*non*−*linearity*_ = 0.52). Specifically, D-dimer levels showed a weak negative correlation with the risk of HS. To be specific, with the increase in D-dimer level by 50 ng/ml, the risk of HS decreased by 1.8% accordingly (RR 0.982, 95%CI 0.920–1.049).

#### Ischemic Stroke

It was observed from Figure 2 that four studies involving 15,699 participants reported the associations between high D-dimer levels and the risk of IS. To be specific, the high D-dimer levels significantly increased the risk of IS by 55% (RR 1.55, 95%CI 1.22–1.98; *I*^2^ 0%). Similarly, the risk of HS increased by 11% with the per-SD increment in D-dimer level (RR 1.11, 95%CI 1.02–1.22; *I*^2^ 0%).

As presented in Figure 3C, there was a linear relationship between D-dimer levels and the risk of IS (*p*_*non*−*linearity*_ = 0.46). Specifically, dose–response analyses on three studies indicated that D-dimer levels exhibited a weak positive correlation with the risk of IS. When the D-dimer level increased by 50 ng/ml, the risk of stroke increased by 0.5% accordingly (RR 1.005, 95%CI 0.924–1.094).

### Post-Stroke or TIA

#### All-Cause Mortality

**Figure 4:**
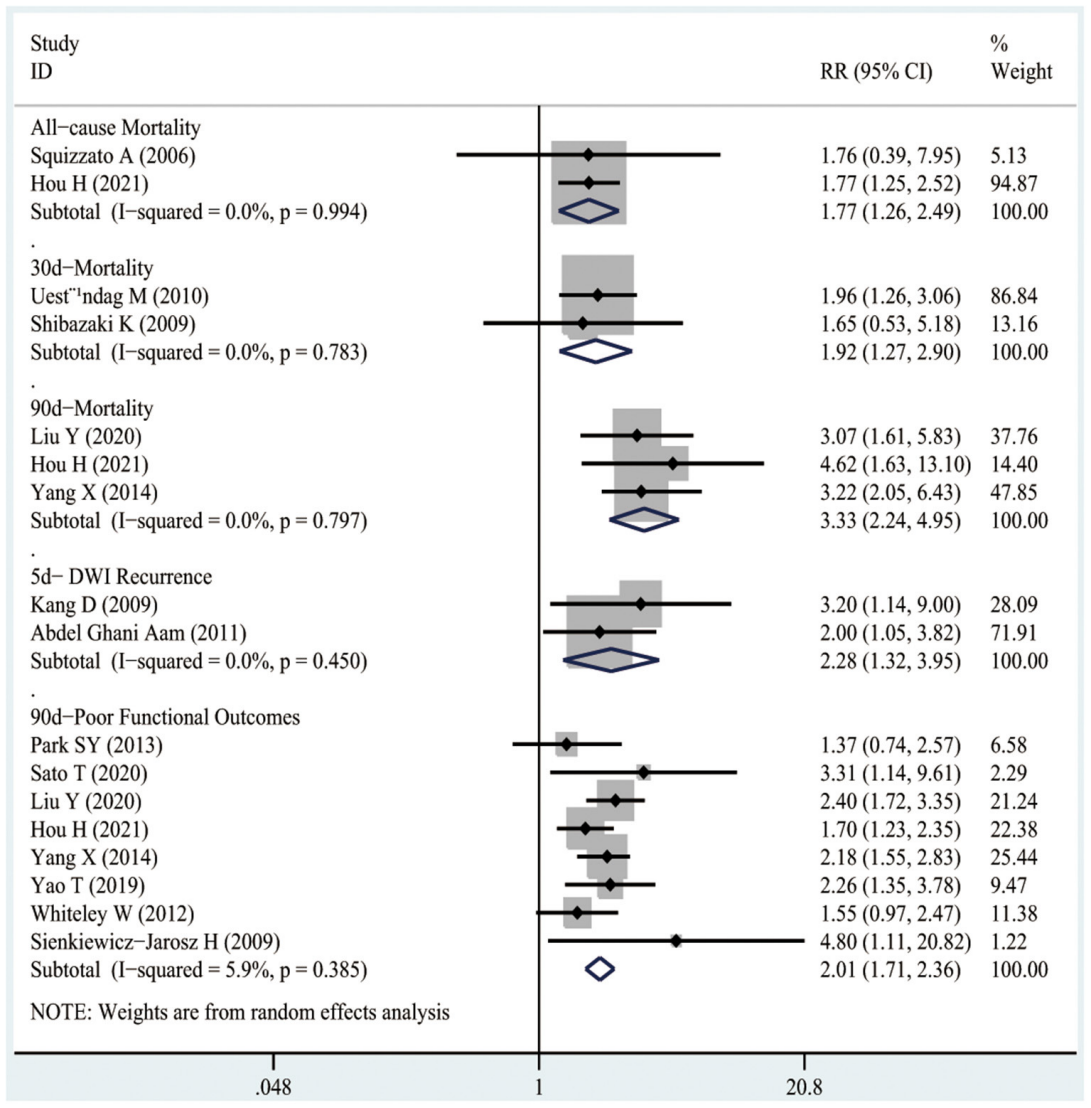
The RRs of high D-dimer levels at admission for the risk of adverse clinical outcomes in patients with AIS or TIA.

Two studies recruiting 10,614 participants mentioned the associations between D-dimer levels on admission and the risk of all-cause mortality in patients with AIS or TIA. As shown in Figure 4, high D-dimer levels on admission were notably associated with an increased risk of all-cause mortality in AIS or TIA patients (RR1.77, 95%CI 1.26–2.49; *I*^2^ 0%).

#### 30-Day Mortality

As presented in Figure 4, two studies involving 426 patients illustrated the associations between D-dimer levels on admission and the risk of 30-day mortality after the occurrence of AIS. As a result, high D-dimer levels on admission were significantly associated with an increased risk of 30-day mortality in AIS patients (RR1.92, 95%CI 1.27–2.9; *I*^2^ 0%).

#### 90-Day Mortality

**Figure 5:**
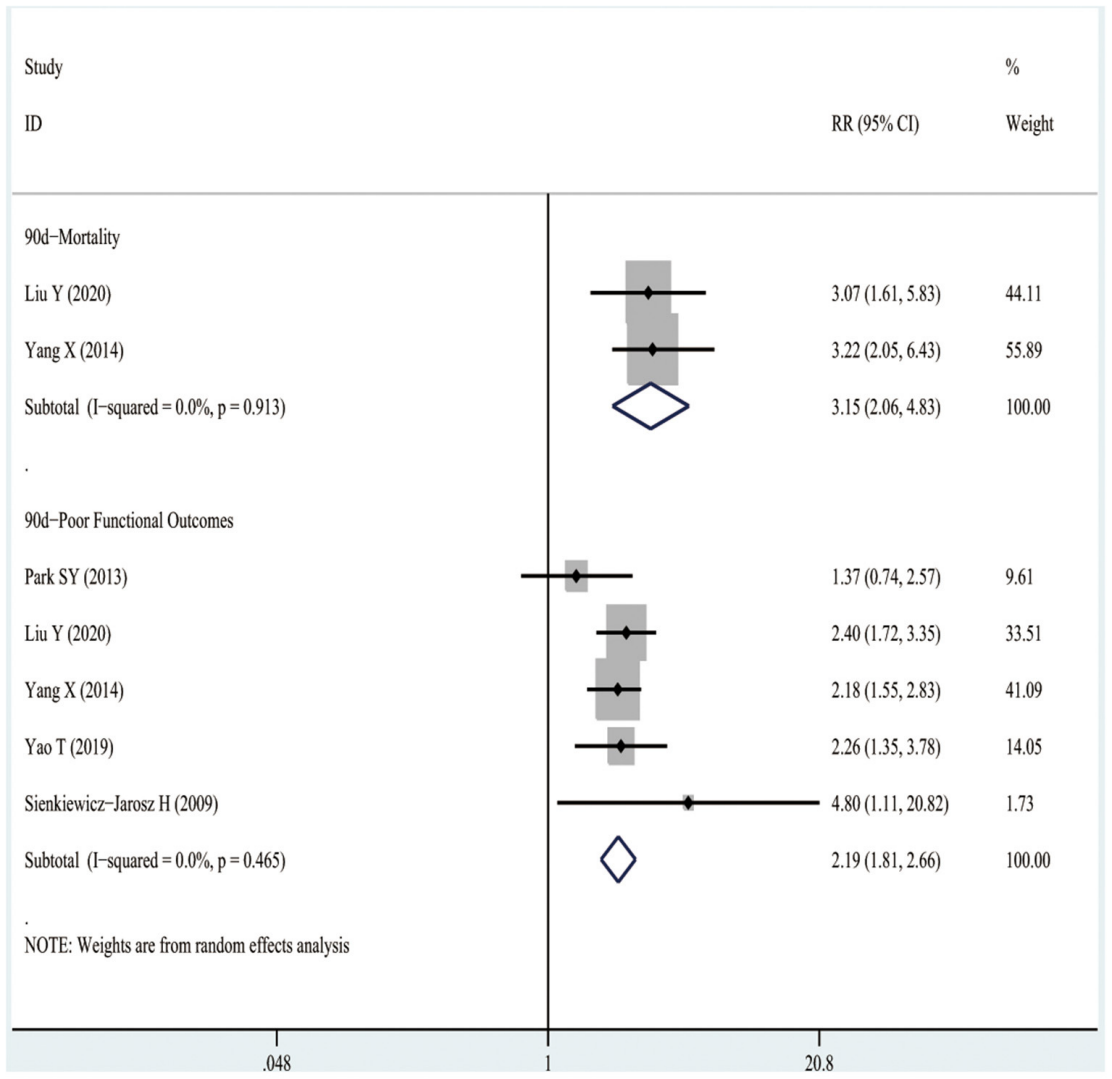
The RRs of high D-dimer levels at admission for the risk of adverse clinical outcomes in patients with AIS.

Figure 4 shows that three studies enrolling 12,206 patients reported the associations between D-dimer levels on admission and the risk of 90-day mortality after the occurrence of AIS or TIA. The results indicated that high D-dimer levels on admission were significantly associated with a higher risk of 90-day mortality after AIS or TIA (RR3.33, 95%CI 2.24–4.95; *I*^2^ 0%). Similarly, even after the TIA population was excluded, high D-dimer levels still increased the risk of 90-day mortality in AIS patients (RR3.15, 95%CI 2.06–4.83; *I*^2^ 0%), as shown in Figure 5.

#### Recurrence

As illustrated in Figure 4, two studies including 203 patients mentioned the relationship between high D-dimer levels on admission and the risk of 5-day DWI recurrence after AIS. It was suggested that high D-dimer levels on admission significantly increased the risk of 5-day DWI recurrence in AIS patients (RR2.28, 95%CI 1.32–3.95; *I*^2^ 0%).

#### 90-Day Poor Functional Outcomes

It was observed from Figure 4 that eight studies involving 13,710 patients reported the relationships between D-dimer levels on admission and the risk of 90-day poor functional outcomes after the occurrence of AIS or TIA. As a result, high D-dimer levels on admission were significantly associated with an increased risk of 90-day poor functional outcomes after AIS or TIA (RR2.01, 95%CI 1.71–2.36; *I*^2^ 0%). Similarly, even after the TIA population was excluded, the high D-dimer levels still increased the risk of 90-day poor functional outcomes in AIS patients (RR2.19, 95%CI 1.81–2.66; *I*^2^ 0%), as shown in Figure 5.

Moreover, the funnel plot (Supplementary Figure 1) showed visual symmetry. No obvious evidence was found upon Begger's-test (*p* = 0.645).

### Subgroup and Sensitivity Analyses

Dose–response analysis on the risk of adverse clinical outcomes after AIS or TIA was not further conducted due to the limited data available. Also, neither subgroup nor sensitivity analysis was carried out because of the limited data.

## Discussion

Results of this meta-analysis suggested that high D-dimer levels were associated with the risk of total stroke and IS, but not with HS. Also, high D-dimer levels on admission were related to the risks of all-cause mortality, 30-day mortality, 90-day mortality, 5-day DWI recurrence, and 90-day poor functional outcomes in patients with AIS or TIA. However, as revealed by dose–response analyses on D-dimer levels and the risk of stroke, there was no significant dose-dependent relationship between D-dimer levels and the risk of stroke.

Although several previous cohort studies reported the associations between D-dimer levels and the risk of stroke, their findings were not completely consistent. For instance, the studies by Wannamethee et al., Folsom et al., and Di Castelnuovo et al. showed that high D-dimer levels were significantly associated with the risk of stroke (8, 9, 25), whereas Zakai et al. reported little evidence supporting the associations between D-dimer levels and the risk of stroke when D-dimer levels were divided into quintiles (26). In addition, D-dimer levels were grouped by different criteria between studies, and such heterogeneities might amplify the effect of D-dimer levels on the risk of stroke. For instance, the study by Wannamethee et al. divided D-dimer levels (60.95–160.77 ng/ml) into tertiles, whereas Zakai divided D-dimer levels (0–900 ng/ml) into quintiles. Therefore, dose–response analysis might contribute to better understanding the effect of D-dimer on stroke. However, based on the limited data available, no relationships between D-dimer levels and the risk of stroke have been found, and more studies are warranted to further validate this result in the future.

The following possible mechanisms may explain the relationships between high D-dimer levels and the risk of stroke or TIA. The increased D-dimer levels may reflect the ongoing thrombosis in cerebral blood vessels (40). Therefore, D-dimer levels may serve as a sign of systemic high blood clotting (27). In addition, it is reported that D-dimer activates the inflammation process, which may involve the activation of monocytes and the release of pro-inflammatory cytokines, such as interleukin-6 (IL-6) (41). Some studies have reported that D-dimer is the most common risk factor for post-stroke venous thrombotic events, which reflects a pre-thrombotic state and increases the susceptibility to major thrombotic events (42).

Noteworthily, our meta-analysis has the following strengths. Firstly, previous meta-analyses have summarized the results simply by high vs. low D-dimer levels, which are not comprehensive enough (10, 43). By contrast, this meta-analysis further elaborated the relationships between D-dimer levels and the risk of stroke through dose–response analyses on the basis of previous studies. Secondly, to ensure the high level of evidence, only prospective studies were enrolled in this meta-analysis. Thirdly, the random-effects model was adopted to synthesize RRs, thus ensuring the stability of our results. Besides, the low heterogeneity between studies further ensured the stability of our study results.

Nonetheless, several limitations should also be noted in this meta-analysis. First of all, based on the current data, the dose–response analyses on the D-dimer levels and the risk of adverse clinical outcomes were not conducted. Similarly, for almost all of the endpoints, subgroup and sensitivity analyses were not performed due to the limited number of studies enrolled. Secondly, the number of the included studies was relatively limited, the results of dose–response analyses were still unstable, and more studies are required for further investigation. Thirdly, the included studies were mainly from Europe, America, and Asia, while relevant studies from other regions were lacking. Fourthly, although the extracted RRs were subjected to multivariate adjustment, some potential confounding factors were not excluded. Last but not least, the study population of this meta-analysis focused on the general population. For other populations with specific diseases such as cancer, diabetes, and vascular disease, the correlations between D-dimer levels and the risk of stroke and clinical outcomes in patients with AIS or TIA remain unknown.

## Conclusions

Based on our results in this meta-analysis, high D-dimer levels may be associated with the risks of total stroke and IS, but not with HS. However, dose–response analyses reveal no obvious evidence for the dose-dependent association between D-dimer levels and the risk of stroke. Besides, high D-dimer levels on admission may be correlated with the risks of adverse clinical outcomes, including all-cause mortality, 5-day recurrence, and 90-day poor functional outcomes, in patients with AIS or TIA. More studies are needed to quantify the effect of D-dimer levels on the risk of stroke or TIA, so as to verify and substantiate this conclusion in the future.

## Data Availability Statement

The original contributions presented in the study are included in the article/Supplementary Material, further inquiries can be directed to the corresponding author.

## Author Contributions

BY participated in the data collection, data review, relevant data extraction, data analysis, statistical analysis, and the writing of the manuscript. TYang, TYan, and XB participated in checking data extraction as well as in the data analysis, statistical analysis, and the writing of the manuscript. WC participated in checking data extraction as well as in the statistical analysis and the writing of the manuscript. All authors saw and approved the final version.

## Conflict of Interest

The authors declare that the research was conducted in the absence of any commercial or financial relationships that could be construed as a potential conflict of interest.

## Supplementary Material

Supplementary Figure 1 The funnel of D-dimer and the risk of poor functional outcomes.

Supplementary Table 1 Quality assessment of the 22 included studies.

## References

[1] Wang W Jiang B Sun H Ru X Sun D Wang L. Prevalence, incidence, and mortality of stroke in china: results from a nationwide population-based survey of 480,687 adults. Circulation. (2017) 135:759–71. 10.1161/CIRCULATIONAHA.116.025250 28052979

[2] Yew KS Cheng EM. Diagnosis of acute stroke. Am Fam Physician. (2015) 91:528–36. 10.3109/9780203008584.007 25884860

[3] Uestuendag M Orak M Gueloglu C Tamam Y Sayhan MB. Plasma D-Dimer levels in acute ischemic stroke: association with mortality, stroke type and prognosis. Nobel Med. (2010) 6:37–42. 10.1002/msj.20185

[4] Adam SS Key NS Greenberg CS. D-dimer antigen: current concepts and future prospects. Blood. (2009) 113:2878–87. 10.1182/blood-2008-06-165845 19008457

[5] Lowe GD. Fibrin D-dimer and cardiovascular risk. Semin Vasc Med. (2005) 5:387–98. 10.1055/s-2005-922485 16302161

[6] Taylor HA Jr. Wilson JG Jones DW Sarpong DF Srinivasan A Garrison RJ. Toward resolution of cardiovascular health disparities in African Americans: design and methods of the Jackson Heart Study. Ethn Dis. (2005) 15(Suppl. 6):S6-4-17. 10.1097/00001648-200509000-00256 16320381

[7] Duprez DA Otvos J Sanchez OA Mackey RH Tracy R Jacobs DR Jr. Comparison of the predictive value of GlycA and other biomarkers of inflammation for total death, incident cardiovascular events, noncardiovascular and noncancer inflammatory-related events, and total cancer events. Clin Chem. (2016) 62:1020–31. 10.1373/clinchem.2016.255828 27173011

[8] Di Castelnuovo A Agnoli C de Curtis A Giurdanella MC Sieri S Mattiello A. Elevated levels of D-dimers increase the risk of ischaemic and haemorrhagic stroke. Findings from the EPICOR Study. Thromb Haemost. (2014) 112:941–6. 10.1160/th14-04-0297 25030937

[9] Folsom AR Gottesman RF Appiah D Shahar E Mosley TH. Plasma d-Dimer and incident ischemic stroke and coronary heart disease: the atherosclerosis risk in communities study. Stroke. (2016) 47:18–23. 10.1161/STROKEAHA.115.011035 26556822 PMC4696899

[10] Zhang J Liu L Tao J Song Y Fan Y Gou M. Prognostic role of early D-dimer level in patients with acute ischemic stroke. PLoS ONE. (2019) 14:e0211458. 10.1371/journal.pone.0211458 30707716 PMC6358072

[11] Wang J Ning R Wang Y. Plasma D-dimer Level, the promising prognostic biomarker for the acute cerebral infarction patients. J Stroke Cerebrovasc Dis. (2016) 25:2011–5. 10.1016/j.jstrokecerebrovasdis.2015.12.031 27234921

[12] Stroup DF Berlin JA Morton SC Olkin I Williamson GD Rennie D. Meta-analysis of observational studies in epidemiology: a proposal for reporting. Meta-analysis of observational studies in epidemiology (MOOSE) group. JAMA. (2000) 283:2008–12. 10.1001/jama.283.15.2008 10789670

[13] Wells GA Shea B O'Connell D Peterson J Welch V Losos M. The Newcastle-Ottawa Scale (NOS) for Assessing the Quality of Nonrandomised Studies in Meta-Analyses. (2014). Available online at: http://www.ohri.ca/programs/clinical_epidemiology/oxford.asp.

[14] Pruance SL Reid JE Grace M Samore M. Hazard ratio in clinical trials. Antimicrob Agents Chemother. (2004) 48:2787–92. 10.1128/AAC.48.8.2787-2792.2004 15273082 PMC478551

[15] Viera AJ. Odds ratios and risk ratios: what's the difference and why does it matter? South Med J. (2008) 101:730–4. 10.1097/SMJ.0b013e31817a7ee4 18580722

[16] Zhang J Yu KF. What's the relative risk? A method of correcting the odds ratio in cohort studies of common outcomes. JAMA. (1998) 280:1690–1. 10.1001/jama.280.19.1690 9832001

[17] Ronksley PE Brien SE Turner BJ Mukamal KJ Ghali WA. Association of alcohol consumption with selected cardiovascular disease outcomes: a systematic review and meta-analysis. BMJ. (2011) 342:d671. 10.1136/bmj.d671 21343207 PMC3043109

[18] Begg CB Mazumdar M. Operating characteristics of a rank correlation test for publication bias. Biometrics. (1994) 50:1088–101. 10.2307/2533446 7786990

[19] Xu C Doi SAR. The robust error meta-regression method for dose-response meta-analysis. Int J Evid Based Healthcare. (2018) 16:138–44. 10.1097/XEB.0000000000000132 29251651

[20] Cheng W Zhang Z Cheng W Yang C Diao L Liu W. Associations of leisure-time physical activity with cardiovascular mortality: a systematic review and meta-analysis of 44 prospective cohort studies. Eur J Prev Cardiol. (2018) 25:1864–72. 10.1177/2047487318795194 30157685

[21] Raffield LM Zakai NA Duan Q Laurie C Smith JD Irvin MR. D-Dimer in African Americans: whole genome sequence analysis and relationship to cardiovascular disease risk in the Jackson heart study. Arterioscler Thromb Vasc Biol. (2017) 37:2220–7. 10.1161/ATVBAHA.117.310073 28912365 PMC5658238

[22] Smith A Patterson C Yarnell J Rumley A Ben-Shlomo Y Lowe G. Which hemostatic markers add to the predictive value of conventional risk factors for coronary heart disease and ischemic stroke? The Caerphilly study. Circulation. (2005) 112:3080–7. 10.1161/CIRCULATIONAHA.105.557132 16286603

[23] Tzoulaki I Murray GD Lee AJ Rumley A Lowe GD Fowkes FG. Relative value of inflammatory, hemostatic, and rheological factors for incident myocardial infarction and stroke: the Edinburgh Artery study. Circulation. (2007) 115:2119–27. 10.1161/CIRCULATIONAHA.106.635029 17404162

[24] Carcaillon L Gaussem P Ducimetière P Giroud M Ritchie K Dartigues JF. Elevated plasma fibrin D-dimer as a risk factor for vascular dementia: the Three-City cohort study. J Thromb Haemost. (2009) 7:1972–8. 10.1111/j.1538-7836.2009.03603.x 19735443

[25] Wannamethee SG Whincup PH Lennon L Rumley A Lowe GD. Fibrin D-dimer, tissue-type plasminogen activator, von Willebrand factor, and risk of incident stroke in older men. Stroke. (2012) 43:1206–11. 10.1161/STROKEAHA.111.636373 22382157

[26] Zakai NA McClure LA Judd SE Kissela B Howard G Safford M. D-dimer and the risk of stroke and coronary heart disease. The reasons for geographic and racial differences in stroke (REGARDS) study. Thromb Haemost. (2017) 117:618–24. 10.1160/TH16-07-0519 28004063 PMC5824689

[27] Zakai NA Olson NC Judd SE Kleindorfer DO Kissela BM Howard G. Haemostasis biomarkers and risk of intracerebral haemorrhage in the reasons for geographic and racial differences in stroke study. Thromb Haemost. (2017) 117:1808–15. 10.1160/TH17-03-0189 28692106 PMC6309529

[28] Squizzato A Ageno W Finazzi S Mera V Romualdi E Bossi A. D-dimer is not a long-term prognostic marker following acute cerebral ischemia. Blood Coagul Fibrinolysis. (2006) 17:303–6. 10.1097/01.mbc.0000224850.57872.d0 16651873

[29] Abdel Ghani AAM Zaitoun AM Gawish HaH Warda MHA. Prognostic value of D-dimer in diffusion weighted-MRI defined early ischemic stroke recurrence. Egypt J Neurol Psychiatry Neurosurg. (2011)48:215–22.

[30] Park SY Kim J Kim OJ Kim JK Song J Shin DA. Predictive value of circulating interleukin-6 and heart-type fatty acid binding protein for three months clinical outcome in acute cerebral infarction: multiple blood markers profiling study. Crit Care. (2013) 17:R45. 10.1186/cc12564 23497639 PMC3672476

[31] Sato T Sato S Yamagami H Komatsu T Mizoguchi T Yoshimoto T. D-dimer level and outcome of minor ischemic stroke with large vessel occlusion. J Neurol Sci. (2020) 413:116814. 10.1016/j.jns.2020.116814 32259707

[32] Liu Y Li F Sun H Sun Y Sun H Zhai Y. Combined prognostic significance of D-dimer level and platelet count in acute ischemic stroke. Thromb Res. (2020) 194:142–9. 10.1016/j.thromres.2020.05.021 32788106

[33] Hou H Xiang X Pan Y Li H Meng X Wang Y. Association of level and increase in D-Dimer with all-cause death and poor functional outcome after ischemic stroke or transient ischemic attack. J Am Heart Assoc. (2021) 10:e018600. 10.1161/JAHA.120.018600 33412918 PMC7955415

[34] Shibazaki K Kimura K Okada Y Iguchi Y Uemura J Terasawa Y. Plasma brain natriuretic peptide as an independent predictor of in-hospital mortality after acute ischemic stroke. Intern Med. (2009) 48:1601–6. 10.2169/internalmedicine.48.2166 19755761

[35] Yang XY Gao S Ding J Chen Y Zhou XS Wang JE. Plasma D-dimer predicts short-term poor outcome after acute ischemic stroke. PLoS ONE. (2014) 9:e89756. 10.1371/journal.pone.0089756 24587013 PMC3933671

[36] Kang DW Yoo SH Chun S Kwon KY Kwon SU Koh JY. Inflammatory and hemostatic biomarkers associated with early recurrent ischemic lesions in acute ischemic stroke. Stroke. (2009) 40:1653–8. 10.1161/STROKEAHA.108.539429 19265045

[37] Yao T Tian BL Li G Cui Q Wang CF Zhang Q. Elevated plasma D-dimer levels are associated with short-term poor outcome in patients with acute ischemic stroke: a prospective, observational study. BMC Neurol. (2019) 19:175. 10.1186/s12883-019-1386-3 31331288 PMC6643313

[38] Whiteley W Wardlaw J Dennis M Lowe G Rumley A Sattar N. The use of blood biomarkers to predict poor outcome after acute transient ischemic attack or ischemic stroke. Stroke. (2012) 43: 86–91 10.1161/STROKEAHA.111.634089 22020034

[39] Sienkiewicz-Jarosz H Gałecka-Wolska M Bidziński A Turzyńska D Sobolewska A Lipska B. Predictive value of selected biochemical markers of brain damage for functional outcome in ischaemic stroke patients. Neurol Neurochir Pol. (2009) 43:126–33. 19484689

[40] Barber M Langhorne P Rumley A Lowe GD Stott DJ. Hemostatic function and progressing ischemic stroke: D-dimer predicts early clinical progression. Stroke. (2004) 35:1421–5. 10.1161/01.STR.0000126890.63512.41 15073383

[41] Robson SC Shephard EG Kirsch RE. Fibrin degradation product D-dimer induces the synthesis and release of biologically active IL-1 beta, IL-6 and plasminogen activator inhibitors from monocytes *in vitro*. Br J Haematol. (1994) 86:322–6. 10.1111/j.1365-2141.1994.tb04733.x 8199021

[42] Simes J Robledo KP White HD Espinoza D Stewart RA Sullivan DR. D-Dimer predicts long-term cause-specific mortality, cardiovascular events, and cancer in patients with stable coronary heart disease: LIPID STudy. Circulation. (2018) 138:712–23. 10.1161/CIRCULATIONAHA.117.029901 29367425

[43] Zhang J Song Y Shan B He M Ren Q Zeng Y. Elevated level of D-dimer increases the risk of stroke. Oncotarget. (2017) 9:2208–19. 10.18632/oncotarget.23367 29416765 PMC5788633

