# Supplementary figures and images for: Relationships Between D-Dimer Levels and Stroke Risk as Well as Adverse Clinical Outcomes After Acute Ischemic Stroke or Transient Ischemic Attack: A Systematic Review and Meta-Analysis
Source: Front Neurol. 2021 Jun 7;12:670730. doi: 10.3389/fneur.2021.670730 (PMC8215146; doi:10.3389/fneur.2021.670730)

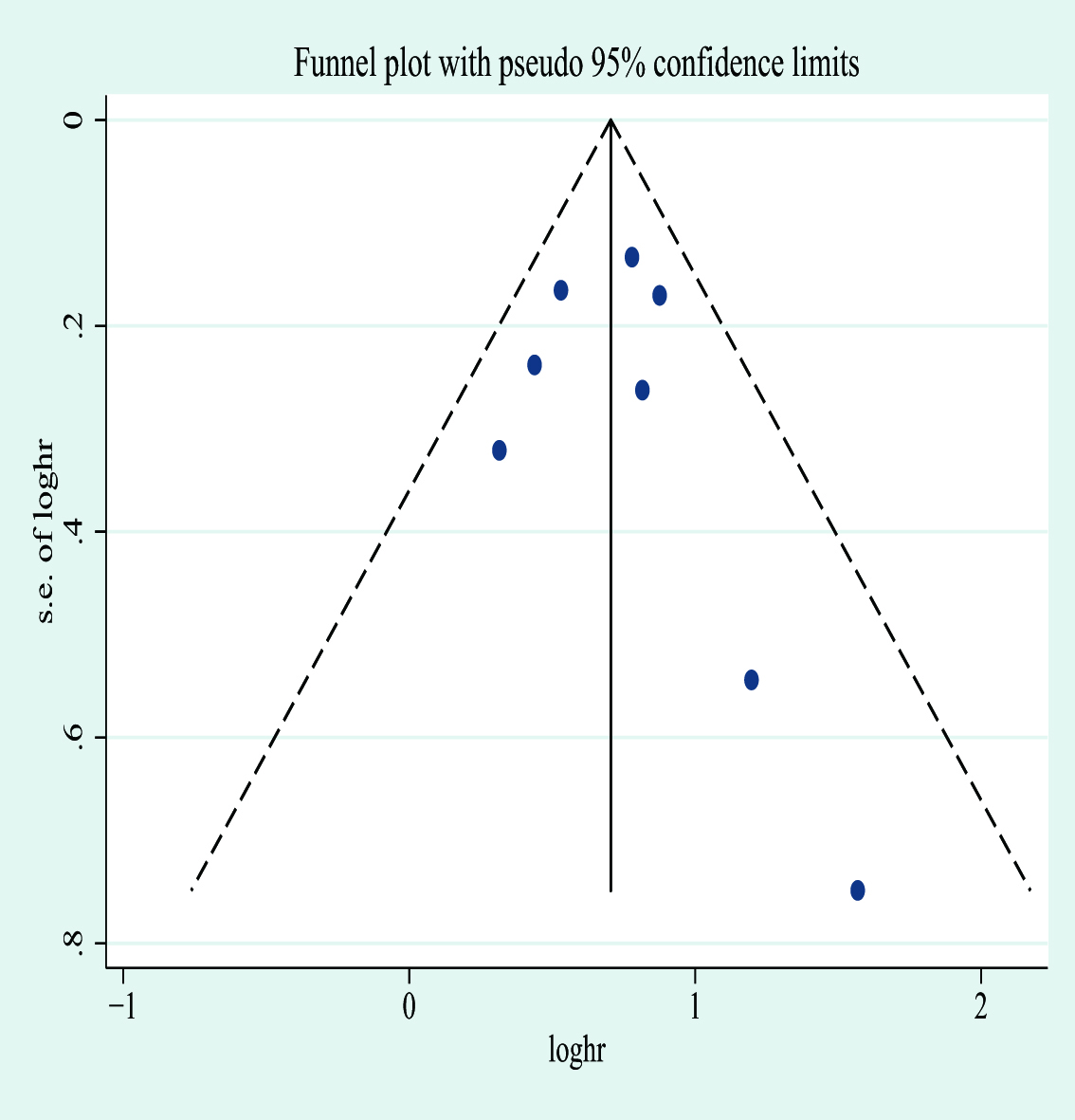

Supplement: Supplementary Figure 1 — The funnel of D-dimer and the risk of poor functional outcomes. [file Image_1.JPEG]
