# Supplementary material for: Relationships Between D-Dimer Levels and Stroke Risk as Well as Adverse Clinical Outcomes After Acute Ischemic Stroke or Transient Ischemic Attack: A Systematic Review and Meta-Analysis
Source: Front Neurol. 2021 Jun 7;12:670730. doi: 10.3389/fneur.2021.670730 (PMC8215146; doi:10.3389/fneur.2021.670730)
Supplement: Supplementary Table 1 — Quality assessment of the 22 included studies. [file Table_1.docx]

| ***Table1.The Detailed Characters of The Included 22 Prospective Studies*** | | | | | | | | | | | |
| --- | --- | --- | --- | --- | --- | --- | --- | --- | --- | --- | --- |
| Author,year | Country | Sample  Size | Female/  Male | Mean  Age | Time of Recruitment | Follow-up | D-Dimer  Range | D-Dimer  Assay | Type of  Stroke | Stroke Status/  Ascertainment | Endpoints |
| ***Before stroke*** |  |  |  |  |  |  |  |  |  |  |  |
| Raffield LM^[21]^  ,2017 | USA | 4,163 | 2585/1578 | 55y | 2000-2004 | 4y | 0.01-11.98ug/ml | ITA | Total  Stroke | Medical Recaords | Total Stroke, All-Cause Mortality |
| Smith Ann^[22]^,  2015 | UK | 2,208 | 0/2182 | 56.9y | 1984-1988 | Median  13y | ＜9ng/ml;9-17ng/ml;＞17ng/ml | ELISA | IS | ICD | IS |
| Tzoulaki L^[23]^,  2007 | UK | 1,592 | 783/809 | 64.9y | NR | Mean 17y | 68-146ng/ml | ELISA | Total  Stroke | Medical Records | Total Stroke |
| Carcaillon L^[24]^  ,2009 | France | 1,254 | 759/495 | 74y | 1999-2001 | 4y | NR | ELISA | IS,HS | CT or MRI | IS,HS |
| Wannamethee SG^[25]^,2012 | UK | 3,358 | 0/3358 | 68.7y | 1978-1980 | Mean 9y | ＜56.8ng/ml;56.8-97.4ng  /ml;＞97.5ng/ml | ELISA | Total  Stroke | CT or MRI | Total Stroke |
| Folsom AR^[9]^,  2016 | USA | 11,415 | 6632/4783 | 59.8y | 1992-1995 | Median  18y | ≤0.14ug/ml;0.15-0.22ug/  ml;0.23-0.32ug/ml;0.33-0.49ug/ml;＞0.49ug/ml | ITA | Total  Stroke,  IS,HS | ICD-9 | Stroke;IS;HS |
| Castelnuovo AD^[8]^,2014 | Italy | 822 | 550/282 | 49.4y | 1993-1998 | 35-71y | ＜100ng/ml;100-127ng  /ml;127.1-166ng/ml;＞166ng/ml | ITA | Total  Stroke,  IS,HS | ICD-10 | Total Stroke;IS;HS |
| Zakai NA(a)^[26]^  ,2017 | USA | 1,180 | 664/516 | 65.4 | 2003-2007 | Median  5.8y | 0-0.22ug/ml;0.23-0.32ug/ml;0.33-0.51ug/ml;0.52-0.89ug/ml;≥0.9ug/ml | ITA | Total  Stroke | Medical  Records | Total Stroke;Per-SD |
| Zakai NA(b)^[27]^  ,2017 | USA | 1,052 | 530/522 | 65.1 | 2003-2007 | Median  5.8y | 0.08-0.29ug/ml;0.29-0.62ug/ml;0.62-2.64ug/ml | ITA | HS | Medical  Records | HS |
| ***After stroke*** |  |  |  |  |  |  |  |  |  |  |  |
| Squizzato A^[28]^,  2006 | Italy | 96 | 54/42 | 74.9y | 1998.1-1999.12 | 61.5months | ≤5ug/ml;0.51-1.5  ug/ml;＞1.5ug/ml | ITA | AIS or  TIA | Post-stroke/  CT | All-cause Mortality |
| Üstündag M^[29]^  ,2010 | Turkey | 91 | 49/42 | 64.5y | 2007.2-2007.12 | 10months | ≤5ug/ml;0.51-1.5  ug/ml;＞1.51ug/ml | ITA | AIS | Post-stroke/  CT or MRI | 30d-Mortality |
| Abdel Ghani  AAM^[30]^,2011 | Egypt | 50 | 17/33 | 60.6y | NR | 5days | NR | ITA | AIS | Post-stroke / CT or MRI | 5d- DWI Recurrence |
| Park SY^[31]^,  2013 | South  Korea | 175 | 80/95 | 66y | 2009.1-2010.6 | 90days | 355-1629ng/ml | ELISA | AIS | Post-stroke / CT or MRI | 90d-Poor Functional Outcomes |
| Sato T^[32]^,2020 | Japan | 130 | 44/86 | 66-81y | 2011.10-2017.2 | 90days | 600-2800ug/l | NR | AIS or  TIA | Post-stroke / CT or MRI | 90d-Poor Functional Outcomes |
| Liu Y^[33]^,2020 | China | 1468 | 508/960 | 64.26y | 2016.4-2019.12 | 1y | ≤0.5mg/l;＞0.5mg/l | ELISA | AIS | Post-stroke / CT or MRI | 90d-Poor Functional Outcomes;90d-Mortality |
| Hou H^[34]^,2021 | China | 10,518 | 3283/7235 | 62.3y | 2015.8-2018.3 | 1y | <0.6ug/ml;6-1ug/ml;1.1-2.0ug/ml; >2.0ug/ml | ITA | AIS or  TIA | Post-stroke / CT or MRI | 90d-Poor Functional Outcomes;90d-Mortality;All-cause Mortaltiy |
| Shibazaki K^[35]^,  2009 | Japan | 335 | 125/210 | 72.3y | 2006.3-2008.4 | 30days | 0-9.5ug/ml | NR | AIS | Post-stroke / CT or MRI | 30 d-Mortality |
| Yang X^[36]^,  2014 | China | 220 | 93/127 | Median  68y | 2011.2-2012.12 | 90days | 0.35-4.62mg/l | ITA | AIS | Post-stroke / MRI | 90d-Poor Functional Outcomes;90d- Mortality |
| Kang D^[37]^,  2009 | Korea | 153 | 54/99 | 64.6y | 2004.12-2006.3 | 5days | 220-1190ng/ml | ELISA | AIS | Post-stroke / MRI | 5d- DWI Recurrence |
| Yao T^[38]^,2019 | China | 877 | 287/600 | Median  64y | 2017.1-2018.8 | 90days | ≤0.24mg/l;0.25-0.56mg/  l;0.57-1.78mg/l;>1.78mg/l | ITA | AIS | Post-stroke / CT or MRI | 90d-Poor Functional Outcomes |
| Whiteley W^[39]^,  2012 | UK | 268 | 156/112 | 74.4y | 2007.3-2009.2 | 90days | 109-440ng/ml | ELISA | AIS | Post-stroke / CT or MRI | 90d-Poor Functional Outcomes |
| Sienkiewicz-Jarosz H^[40]^,2009 | Poland | 54 | 27/27 | 73.3y | NR | 90days | NR | ITA | AIS | Post-stroke / CT or MRI | 90d-Poor Functional Outcomes |

AIS , acute ischemic stroke.TIA,transient ischemic attack.CT, computed tomographic. ITA: immunoturbidimetric assay. MRI, magnetic resonance imagin.ELISA: enzyme-linked immunosorbent assay Long-term Mortality,motality over 3 months.30d-mortaliy,mortality at 30day.30d-Poor Functional Outcomes, poor functional outcome at 30 days.90d-Poor Functional Outcomes, poor functional outcome at 90 days. 5d-DWI recurrence,recurrence on 5-day diffusion-weighted.HS, haemorrhagic stroke.IS,ischemic stroke.ICD-9, International Classification of Diseases, Ninth Revision. ICD-10, ICD-9, International Classification of Diseases, tenth Revision.Per-SD,Per standard deviation Increment.
