# Supplementary material for: Relationships Between D-Dimer Levels and Stroke Risk as Well as Adverse Clinical Outcomes After Acute Ischemic Stroke or Transient Ischemic Attack: A Systematic Review and Meta-Analysis
Source: Front Neurol. 2021 Jun 7;12:670730. doi: 10.3389/fneur.2021.670730 (PMC8215146; doi:10.3389/fneur.2021.670730)
Supplement: Supplementary file 3 [file Presentation_1.docx]

**Pubmed**

1. Search: (((((D-dimer fibrin[Title/Abstract]) OR (D-dimer fragments[Title/Abstract])) OR (fibrin fragment D1 dimer[Title/Abstract])) OR (fibrin fragment DD[Title/Abstract])) OR (D-dimer[Title/Abstract])) OR (fibrin fragment D-dimer[Title/Abstract])

2. Search: ("Stroke"[Mesh]) OR ((((((((((((((((((((((((((((Strokes[Title/Abstract]) OR (Cerebrovascular Accident[Title/Abstract])) OR (Cerebrovascular Accidents[Title/Abstract])) OR (CVA (Cerebrovascular Accident)[Title/Abstract])) OR (CVAs (Cerebrovascular Accident)[Title/Abstract])) OR (Cerebrovascular Apoplexy[Title/Abstract])) OR (Apoplexy, Cerebrovascular[Title/Abstract])) OR (Vascular Accident, Brain[Title/Abstract])) OR (Brain Vascular Accident[Title/Abstract])) OR (Brain Vascular Accidents[Title/Abstract])) OR (Vascular Accidents, Brain[Title/Abstract])) OR (Cerebrovascular Stroke[Title/Abstract])) OR (Cerebrovascular Strokes[Title/Abstract])) OR (Stroke, Cerebrovascular[Title/Abstract])) OR (Strokes, Cerebrovascular[Title/Abstract])) OR (Apoplexy[Title/Abstract])) OR (Cerebral Stroke[Title/Abstract])) OR (Cerebral Strokes[Title/Abstract])) OR (Stroke, Cerebral[Title/Abstract])) OR (Strokes, Cerebral[Title/Abstract])) OR (Stroke, Acute[Title/Abstract])) OR (Acute Stroke[Title/Abstract])) OR (Acute Strokes[Title/Abstract])) OR (Strokes, Acute[Title/Abstract])) OR (Cerebrovascular Accident, Acute[Title/Abstract])) OR (Acute Cerebrovascular Accident[Title/Abstract])) OR (Acute Cerebrovascular Accidents[Title/Abstract])) OR (Cerebrovascular Accidents, Acute[Title/Abstract]))

3. Search: (((((("Ischemic Attack, Transient"[Mesh]) OR (Transient Ischemic Attack[Title/Abstract])) OR (Attack, Transient Ischemic[Title/Abstract])) OR (Attacks, Transient Ischemic[Title/Abstract])) OR (Ischemic Attacks, Transient[Title/Abstract])) OR (Transient Ischemic Attacks[Title/Abstract])) OR (TIA[Title/Abstract])

4.2 or 3

5.1 and 4 773

**Embase**

1. 'd dimer'/exp

2. 'crosslinked fibrin degradation product':ab,ti OR 'fibrin degradation product d dimer':ab,ti OR 'd dimer':ab,ti

3.1 or 2

4. 'cerebrovascular accident'/exp

5. 'accident, cerebrovascular':ab,ti OR 'acute cerebrovascular lesion':ab,ti OR 'acute focal cerebral vasculopathy':ab,ti OR 'acute stroke':ab,ti OR 'apoplectic stroke apoplexia':ab,ti OR apoplexia:ab,ti OR apoplexy:ab,ti OR 'blood flow disturbance, brain':ab,ti OR 'brain accident':ab,ti OR 'brain attack':ab,ti OR 'brain blood flow disturbance':ab,ti OR 'brain insult':ab,ti OR 'brain insultus':ab,ti OR 'brain ischaemic attack':ab,ti OR 'brain ischemic attack':ab,ti OR 'brain vascular accident':ab,ti OR 'cerebral apoplexia':ab,ti OR 'cerebral insult':ab,ti OR 'cerebral stroke':ab,ti OR 'cerebral vascular accident':ab,ti OR 'cerebral vascular insufficiency':ab,ti OR 'cerebro vascular accident':ab,ti OR 'cerebrovascular arrest':ab,ti OR 'cerebrovascular failure':ab,ti OR 'cryptogenic stroke':ab,ti OR 'cerebrovascular injury':ab,ti OR 'cerebrovascular insufficiency':ab,ti OR 'cerebrovascular insult':ab,ti OR 'cerebrum vascular accident':ab,ti OR cva:ab,ti OR 'ischaemic cerebral attack':ab,ti OR 'ischaemic seizure':ab,ti OR 'ischemic cerebral attack':ab,ti OR 'ischemic seizure':ab,ti OR stroke:ab,ti

6. 4 or 5

7. 'transient ischemic attack'/exp

8. 'brain transient ischaemic attack':ab,ti OR 'cerebral ischaemia, transient':ab,ti OR 'brain transient ischemic attack':ab,ti OR 'cerebral ischemia, transient':ab,ti OR 'circulatory epilepsy':ab,ti OR 'epilepsy circulatory':ab,ti OR 'ischaemic attack':ab,ti OR 'ischaemic attack, transient':ab,ti OR 'ischemic attack':ab,ti OR 'ischemic attack, transient':ab,ti OR tia:ab,ti OR 'transient cerebral ischemia':ab,ti OR 'transient brain ischemia':ab,ti OR 'transient cerebral ischaemia':ab,ti OR 'transient ischaemic attack':ab,ti OR 'transient ischaemic seizure':ab,ti OR 'transient ischemic seizure':ab,ti OR 'transient brain ischaemia':ab,ti

9.7 or 8

10.6 or 9

11.3 and 10 2063

**Cochrane**

1. (d dimer):ti,ab,kw OR (d-dimer):ti,ab,kw

2. MeSH descriptor: [Stroke] explode all trees

3. (Acute Stroke):ti,ab,kw OR (Strokes, Acute):ti,ab,kw OR (Acute Cerebrovascular Accident):ti,ab,kw OR (Cerebrovascular Accident, Acute):ti,ab,kw OR (Cerebrovascular Accidents, Acute):ti,ab,kw

4. (Acute Strokes):ti,ab,kw OR (Acute Cerebrovascular Accidents):ti,ab,kw OR (Stroke, Acute):ti,ab,kw OR (Cerebrovascular Strokes):ti,ab,kw OR (Stroke, Cerebral):ti,ab,kw

5. (Cerebrovascular Stroke):ti,ab,kw OR (Cerebrovascular Accident):ti,ab,kw OR (Brain Vascular Accident):ti,ab,kw OR (Strokes, Cerebral):ti,ab,kw OR (Apoplexy, Cerebrovascular):ti,ab,kw

6. (CVA (Cerebrovascular Accident)):ti,ab,kw OR (Vascular Accident, Brain):ti,ab,kw OR (Brain Vascular Accidents):ti,ab,kw OR (Cerebral Stroke):ti,ab,kw OR (Vascular Accidents, Brain):ti,ab,kw

7. (Apoplexy):ti,ab,kw OR (CVAs (Cerebrovascular Accident)):ti,ab,kw OR (Strokes):ti,ab,kw OR (Cerebrovascular Accidents):ti,ab,kw OR (Strokes, Cerebrovascular):ti,ab,kw

8. (Cerebral Strokes):ti,ab,kw OR (Stroke, Cerebrovascular):ti,ab,kw OR (Cerebrovascular Apoplexy):ti,ab,kw

9. #2 or #3 or #4 or #5 or #6 or #7 or #8

10. MeSH descriptor: [Ischemic Attack, Transient] explode all trees

11. (Transient Ischemic Attacks, Crescendo):ti,ab,kw OR (Crescendo Transient Ischemic Attacks):ti,ab,kw OR (Transient Ischemic Attack, Brainstem):ti,ab,kw OR (Transient Ischemic Attack, Brain Stem):ti,ab,kw OR (Brainstem Ischemia, Transient):ti,ab,kw

12. (Brain Stem Ischemia, Transient):ti,ab,kw OR (Transient Brainstem Ischemia):ti,ab,kw OR (Ischemias, Transient Brainstem):ti,ab,kw OR (Brainstem Ischemias, Transient):ti,ab,kw OR (Brainstem Transient Ischemic Attack):ti,ab,kw

13. (Brain Stem Transient Ischemic Attack):ti,ab,kw OR (Ischemia, Transient Brainstem):ti,ab,kw OR (Attack, Transient Ischemic):ti,ab,kw OR (Transient Ischemic Attacks):ti,ab,kw OR (Ischemic Attacks, Transient):ti,ab,kw

14. (Brain TIA):ti,ab,kw OR (TIAs (Transient Ischemic Attack)):ti,ab,kw OR (Attacks, Transient Ischemic):ti,ab,kw OR (TIA, Brain):ti,ab,kw OR (Transient Ischemic Attack):ti,ab,kw

15. (TIA (Transient Ischemic Attack)):ti,ab,kw OR (Transient Ischemic Attack, Carotid Circulation):ti,ab,kw OR (Carotid Circulation Transient Ischemic Attack):ti,ab,kw OR (Vertebrobasilar Circulation Transient Ischemic Attack):ti,ab,kw OR (Transient Ischemic Attack, Vertebrobasilar Circulation):ti,ab,kw

16. (Posterior Circulation Transient Ischemic Attack):ti,ab,kw OR (Transient Ischemic Attack, Posterior Circulation):ti,ab,kw OR (Cerebral Ischemias, Transient):ti,ab,kw OR (Cerebral Ischemia, Transient):ti,ab,kw OR (Transient Cerebral Ischemia):ti,ab,kw

17. (Transient Cerebral Ischemias):ti,ab,kw OR (Ischemia, Transient Cerebral):ti,ab,kw OR (Anterior Circulation Transient Ischemic Attack):ti,ab,kw OR (Transient Ischemic Attack, Anterior Circulation):ti,ab,kw OR (Ischemias, Transient Cerebral):ti,ab,kw

18. #10 or#11 or #12 or #13 or #14 or #15 or #16 or #17

19.#9 or #19

20. #1 and #19 596
